# Expressions of heat shock protein 90, inducible nitric oxide synthase, and vascular endothelial growth factor in the skin of diabetic rats

**DOI:** 10.14202/vetworld.2021.1804-1807

**Published:** 2021-07-13

**Authors:** Khaled Z. Alawneh, Liqaa A. Raffee, Musa A. Alshehabat, Ahed Jumah Alkhatib

**Affiliations:** 1 Department of Radiology, Faculty of Medicine, Jordan University of Science and Technology, Irbid 22110, Jordan; 2 Department of Accident and Emergency, Faculty of Medicine, Jordan University of Science and Technology, Irbid 22110, Jordan; 3 Department of Veterinary Clinical Sciences, Faculty of Veterinary Medicine, Jordan University of Science and Technology, Irbid 22110, Jordan; 4 Department of Legal Medicine, Faculty of Medicine, Jordan University of Science and Technology, Irbid 22110, Jordan

**Keywords:** diabetic foot, endocrine system, vascular injury, wound healing

## Abstract

**Background and Aim:**

Chronic skin ulceration is a common and painful condition that affects about 15% of patients with diabetes worldwide. The aim of this study was to evaluate the expressions of heat shock protein 90 (HSP 90), inducible nitric oxide synthase (iNOS), and vascular endothelial growth factor (VEGF) in the skin of diabetic rats.

**Materials and Methods:**

A total of 20 rats were divided randomly into two equal groups. Diabetes mellitus (DM) was induced in the rats of Group 2, whereas the rats in Group 1 were kept healthy and served as control. DM was induced by a single intraperitoneal injection of alloxan monohydrate at 120 mg/kg. Rats were considered diabetic if the blood glucose level was above 200 mg/dL. After induction of DM, the rats were monitored daily for 28 days. On day 28, the rats were humanely euthanized, and full-thickness skin punch biopsy was obtained from the dorsal side of the thoracolumbar region. Indirect immunoperoxidase staining was used to evaluate the expressions of HSP 90, iNOS, and VEGF in the skin tissue specimens.

**Results:**

The expressions of HSP 90, iNOS, and VEGF in the skin were significantly higher in diabetic rats than in the control rats. On day 28 in diabetic rats, a positive correlation (r=0.65, p=0.01) was detected between mean blood glucose level and the expression levels of HSP 90, iNOS, and VEGF.

**Conclusion:**

The results of this study indicated that DM upregulated the expressions of HSP 90, iNOS, and VEGF in the skin tissues of diabetic rats and may impact the healing of skin wounds. However, this study was preliminary and further studies to investigate this relationship are warranted.

## Introduction

Diabetes mellitus (DM) can result in serious complications, including cardiovascular disease, chronic skin ulceration, poor wound healing, cataract, retinopathy, nephropathy, and polyneuropathy [1-6]. Chronic skin ulceration, such as diabetic foot ulcer, is a common and painful condition that affects about 15% of patients with DM worldwide [7]. Although the exact mechanisms of impaired wound healing and development of diabetic foot in patients with DM are not fully understood, results of recent clinical studies have indicated involvement of local sepsis, epithelial damage, immune system impairment, and local ischemia secondary to poor vascular flow as the potential mechanisms [7-10]. Moreover, advanced glycation end products, oxidative stress, and low-grade inflammation have been reported to mediate hyperglycemia-induced cell dysfunction in patients with DM [7-9].

The important roles of heat shock proteins (HSPs) 47 and 70 in the wound healing process of patients with DM have been proposed previously [7-9]. Increased expression levels and plasma concentrations of HSP 47 and HSP 70 have been found in the wounds of diabetic animals [7-9]. The roles of these HSPs (47 and 70) have been extensively studied, but there is a lack of research on the role of HSP 90 in the wound healing of diabetic animals. Inducible nitric oxide synthase (iNOS) has been found to play a major role in posttranslational modification, which triggers insulin resistance in obesity and aging [10]. NO, which is catalyzed by iNOS and is a potent oxidant, has been found to modulate insulin secretion, angiogenesis, nociception, inflammation, and pain [11-15]. During wound healing in patients with DM, excess levels of NO and other oxygen free radicals, such as peroxynitrite and peroxynitrous acid, result in severe endothelial damage and further matrix destruction [15]. Furthermore, the expression of iNOS has been suggested to be upregulated by various insulin resistance inducers, including pro-inflammatory cytokines, such as vascular endothelial growth factor (VEGF) [16]. VEGF is considered an important signal protein that is produced by cells to stimulate angiogenesis, particularly in injured or healing tissues [16]. Locally released VEGF in injured tissues has been found to promote wound healing and reduce the risk of diabetic foot ulcers [15].

The hypothesis in this study was that the expressions of HSP 90, iNOS, and VEGF were enhanced in the dermal tissues of diabetic rats and that the interactions among these molecules may explain some of the complex mechanisms of wound healing in patients with DM. Therefore, the objective of this study was to determine the expression levels of HSP 90, iNOS, and VEGF in the skin of rats with alloxan-induced diabetes.

## Materials and Methods

### Ethical approval

This study was approved by the Ethics Committee of the Jordan University of Science and Technology (ACUC number 16-03-03-36).

### Study period and locations

This study was performed from June to September 2015. All experimental procedures were performed at the Animal House of Jordan University of Science and Technology.

### Animals

Twenty adult albino rats weighing approximately 150-180 g were randomly allocated into the following two groups (10 each): Control group (Group 1) and experimental group (Group 2). All rats were housed individually in cages and offered feed and fresh drinking water *ad libitum*. The rats were maintained at room temperature (23-24^o^C) with 12 h day and night light cycles.

### Induction of DM

DM was induced by a single intraperitoneal injection of 120 mg/kg of alloxan monohydrate (Sigma, USA). After alloxan injection, blood glucose level was measured using a blood glucose meter (Glucocheck, South Africa) once daily for 48 h and weekly thereafter for the duration of the experiment. All rats developed hyperglycemia (blood glucose of ≥200 mg/dL) and were considered diabetic. On day 28 of the experiment, all rats were humanely euthanized by isoflurane overdose in a glass chamber. Immediately after euthanasia, full-thickness skin samples were harvested from the dorsal thoracolumbar area using skin punch biopsy. Skin specimens were placed in 10% buffered formalin.

### Immunohistochemistry

Skin tissue specimens were processed using a tissue processor (Leica Biosystems, USA). Briefly, tissue sections were deparaffinized in an oven at 65°C for 1 h. The sections were then passed into solutions, from xylene to distilled water. To minimize or inhibit endogenous peroxidase activity, the sections were treated with 1% hydrogen peroxide in absolute methanol for 20 min. To minimize or prevent nonspecific binding, the tissue sections were washed with phosphate-buffered saline (PBS, pH 7.2-7.4) for 5 min and incubated with 1% bovine serum albumin for 30 min. During that time, the primary antibody and other immunohistochemistry reagents were prepared and brought to room temperature (23-24^o^C). Monoclonal antibodies against HSP 90, iNOS, and VEGF were prepared (1:100) and incubated with the tissue sections for 1 h in a humid chamber. Thereafter, the tissue sections were washed with PBS for 5 min and incubated with secondary biotinylated antibodies for 20 min. The sections were then washed again with PBS for 5 min and incubated with streptavidin-conjugated with horseradish peroxidase enzyme for 20 min. Thereafter, the sections were washed again with PBS for 5 min. Finally, immunohistochemical reaction was assessed by incubation with diaminobenzidine until the development of a brown reaction followed by washing of the sections with tap water to stop the reaction. The sections were counterstained with hematoxylin for 30 s, washed with water, dehydrated, and mounted with a medium. The size of the tissue specimens for immunohistochemistry staining was 1×1×1 mm.

### Statistical analysis

Data on HSP 90, iNOS, and VEGF were compared between groups using independent Student’s t-test. Levene’s test was used to assess the equality of variances. The expressions of HSP 90, iNOS, and VEGF were assessed by Adobe Photoshop Software version 7.2 (Adobe Systems Inc., California, USA). Photomicrographs of the tissue sections were captured and divided into pixels, which appeared in blue and brown; the brown color represented the marker. The number of brown pixels was computed and expressed as a ratio to the total number of blue and brown pixels. Descriptive statistics for HSP 90, iNOS, and VEGF for each group were presented as mean ± SD. Correlation analysis between the mean blood glucose level on day 28 and the expression levels of HSP 90, iNOS, and VEGF in diabetic rats was performed using Pearson’s correlation. Statistical significance was accepted at p<0.05. All statistical analyses were performed using SPSS software version 19.0 (IBM Statistical Package, USA).

## Results

**Figure-1:**
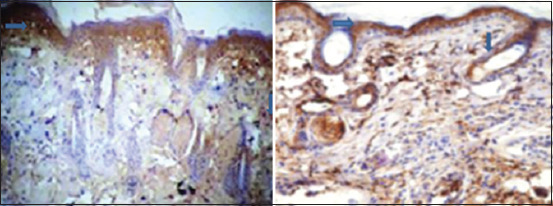
Immunohistochemistry image showing the extent HSP 90 expression in skin of diabetic rats (left) and control rats (right). Arrows point at brown color indicating HSP 90 (40×). There was a significant difference in the expression of HSP 90 in the skin of control rats when compared to that in alloxan-induced diabetic rats. HSP 90=Heat shock protein 90.

**Figure-2:**
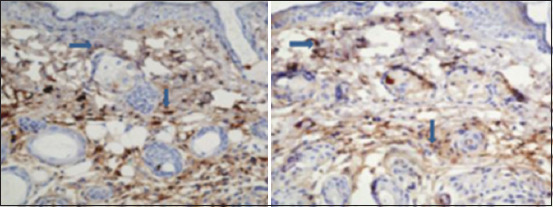
Immunohistochemistry image showing the extent of iNOS expression in skin of diabetic rats (left) and control rats (right). Arrow points at brown color indicate iNOS (40×). There was a significant difference in the expression of iNOS in the skin of control rats when compared to that in alloxan-induced diabetic rats. iNOS=Inducible nitric oxide synthase.

**Figure-3:**
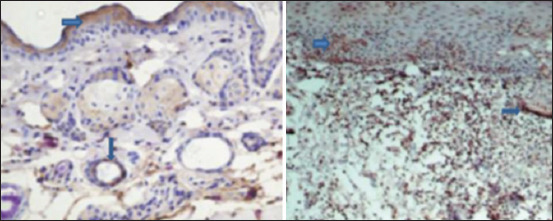
Immunohistochemistry image showing the extent of VEGF expression in skin of diabetic rats (left) and control rats (right). Arrows point at brown color indicating of VEGF (40×). There was a significant difference in the expression of VEGF in the skin of control rats when compared that in alloxan-induced diabetic rats. VEGF=Vascular endothelial growth factor.

All rats in Group 2 developed DM and maintained an average blood glucose level of ≥200 mg/dL throughout the study period. Table-1 presents the values of HSP 90, iNOS, and VEGF expressions in the skin of rats with alloxan-induced DM. A positive correlation (r=0.65, p=0.01) was detected between the mean blood glucose level on day 28 and the expression levels of HSP 90, iNOS, and VEGF in diabetic rats. There was a significant difference in the skin expressions of HSP 90, iNOS, and VEGF between Groups 1 and 2 (Figures-1-3).

**Table-1:** Expression of HSP 90, iNOS, and VEGF in skin of alloxan-induced diabetic rats (values are expressed in mean±SD based on descriptive analysis using pixel count on Photoshop of immunohistochemistry images).

Parameter	Control	Diabetic rats	p-value*
HSP 90	0.09±0.02	0.16±0.05	0.01
iNOS	0.04±0.01	0.48±0.18	0.02
VEGF	0.25±0.21	0.51±0.02	0.05

* Using independent Student’s t-test. HSP 90=Heat shock protein 90, iNOS=Inducible nitric oxide synthase, VEGF=Vascular endothelial growth factor, SD=Standard deviation

## Discussion

This study was carried out to evaluate the expressions of biomarkers HSP 90, iNOS, and VEGF in the skin of normal rats and rats with alloxan-induced diabetes. The results indicated that the skin obtained from diabetic rats had significantly higher expressions of HSP 90, iNOS, and VEGF biomarkers, compared with those in the control group. Overexpression of the biomarkers HSP 90, iNOS, and VEGF in the skin of diabetic rats suggested that the cellular protective response of body is initiated by providing more factors that are required for cutaneous tissue healing and remodeling.

In this study, the dorsal thoracolumbar skin area was selected because it provides easy access and has a larger area for sample collection, compared with the foot in rats [17]. Recently, rapid accumulation of HSP 90a in response to tissue injury in different body cells has been suggested [18]. Moreover, secretion of HSP 90a by keratinocytes at the wound edge to enhance reepithelialization and, eventually, wound closure was reported [19,20]. In fact, wound healing has been observed to accelerate following topical application of recombinant HSP 90a [19-21].

iNOS is known to be expressed in almost all cell types, including the skin [22]. iNOS was suggested to be required for wound healing, and lack of iNOS was reported to retard macrophage invasion and impaired wound healing [23]. Moreover, topical application of recombinant matricellular protein angiopoietin-like 4 was shown to accelerate wound healing by promoting reepithelialization and upregulating iNOS in the epithelium of diabetic mice [24,25]. Furthermore, a pro-angiogenic response through upregulation of VEGF was suggested to promote the wound healing process in diabetic rats [26].

## Conclusion

The results of this study indicated that DM upregulated the expressions of HSP 90, iNOS, and VEGF in the skin tissues of diabetic rats and may impact wound healing. Although the immunohistochemistry data provided in this study may have been insufficient for a complete understanding of the complex relationships between the different parameters and DM, the results may be considered preliminary and may shed some light on the advancement of our understanding of the roles of these proteins in wound healing in patients with DM. Further studies are warranted to investigate the exact mechanisms of these relationships.

## Authors’ Contributions

KZA, LAR, and AJA: Designed the study, performed the laboratory analysis, and collected data. MAA: Performed the statistical analysis and reviewed the manuscript. All authors read and approved the final manuscript.
